# Organ-specific expression and epigenetic traits of genes encoding digestive enzymes in the lance-leaf sundew (*Drosera adelae*)

**DOI:** 10.1093/jxb/eraa560

**Published:** 2020-11-28

**Authors:** Naoki Arai, Yusuke Ohno, Shinya Jumyo, Yusuke Hamaji, Takashi Ohyama

**Affiliations:** 1 Major in Integrative Bioscience and Biomedical Engineering, Graduate School of Science and Engineering, Waseda University, Shinjuku-ku, Tokyo, Japan; 2 Department of Biology, Faculty of Education and Integrated Arts and Sciences, Waseda University, Shinjuku-ku, Tokyo, Japan; 3 Swedish University of Agricultural Sciences, Sweden

**Keywords:** Carnivorous plants, defense-related proteins, DEMETER, DNA methylation, *Drosera*, epigenetic regulation, organ-specific DNA demethylation, sundew, tissue-specific DNA demethylation

## Abstract

Over the last two decades, extensive studies have been performed at the molecular level to understand the evolution of carnivorous plants. As fruits, the repertoire of protein components in the digestive fluids of several carnivorous plants have gradually become clear. However, the quantitative aspects of these proteins and the expression mechanisms of the genes that encode them are still poorly understood. In this study, using the Australian sundew *Drosera adelae,* we identified and quantified the digestive fluid proteins. We examined the expression and methylation status of the genes corresponding to major hydrolytic enzymes in various organs; these included thaumatin-like protein, S-like RNase, cysteine protease, class I chitinase, β-1, 3-glucanase, and hevein-like protein. The genes encoding these proteins were exclusively expressed in the glandular tentacles. Furthermore, the promoters of the β-1, 3-glucanase and cysteine protease genes were demethylated only in the glandular tentacles, similar to the previously reported case of the S-like RNase gene *da-I*. This phenomenon correlated with high expression of the DNA demethylase DEMETER in the glandular tentacles, strongly suggesting that it performs glandular tentacle-specific demethylation of the genes. The current study strengthens and generalizes the relevance of epigenetics to trap organ-specific gene expression in *D. adelae*. We also suggest similarities between the trap organs of carnivorous plants and the roots of non-carnivorous plants.

## Teaser

Lance-leaf sundew provides an intriguing example of organ-specific DNA demethylation, which allows β-1, 3-glucanase, cysteine protease, and S-like RNase genes to be exclusively expressed in the glandular tentacles.

## Introduction

Carnivory is not restricted to animals. Some plants also behave like ‘flesh eaters’ and are referred to as carnivorous plants (Charles Darwin originally called them ‘insectivorous plants’: Darwin, 1875; Lloyd, 1942; Juniper *et al.*, 1989). Carnivorous plants have evolved the ability to trap and digest prey, such as arthropods and small animals, and absorb nutrients from the resulting digest. This ability has allowed them to grow in nutrient-deficient habitats. The carnivorous plants seem to have emerged between 1.9 and 95.1 million years ago (Sadowski *et al.*, 2015; Fleischmann *et al.*, 2018). They are thought to have evolved independently at least nine or ten times in five orders of flowering plants, namely Lamiales, Ericales, Caryophyllales, Oxalidales and Poales (Albert *et al.*, 1992; Givnish, 2015; Fleischmann *et al.*, 2018). Carnivorous plants presently account for at least ~600 species in ~20 genera, ~12 families and five orders of angiosperms (Ellison and Gotelli, 2009; Givnish, 2015).

Carnivorous plants have elaborate leaves or stolons that function as traps, which are grouped into five types (Darwin, 1875; Lloyd, 1942; Juniper *et al.*, 1989; Król *et al.*, 2012; Ellison and Adamec, 2018). These include adhesive traps [e.g. *Drosera* (sundews) and *Pinguicula* (butterworts)], snap traps [*Dionaea* (Venus flytrap) and *Aldrovanda* (waterwheel plant)], pitfall traps [e.g. *Nepenthes* (tropical pitcher plants), *Sarracenia* (American pitcher plants) and *Cephalotus* (Albany pitcher plant)], suction traps [*Utricularia* (bladderworts)], and lobster pot traps [*Genlisea* (corkscrew plants)]. There are two long-standing questions for these plants: how did their ancestors become equipped with the trap leaves and how did they acquire the mechanisms of prey digestion (Darwin, 1875; Lloyd, 1942; Juniper *et al.*, 1989; Renner and Specht, 2013; Ellison and Adamec, 2018; Pavlovič and Mithöfer, 2019)? Recent studies have gradually begun to provide answers to these questions.

Trap leaf acquisition may have originated in hairy leaves and ‘foliar feeding’ in non-carnivorous plants (Fernández and Eichert, 2009; Fernández and Brown, 2013). Hairy leaves can hold raindrops that capture insects by the surface tension of water. Some insects could drown, rot, and finally release nutrients, and this phenomenon may have caused the evolution of the leaves of the ancestors of carnivorous plants. Phylogenetic data suggest that the adhesive trap is the origin of most carnivorous plants (Müller *et al.*, 2004; Heubl *et al.*, 2006). Various trap leaves are thought to have been formed by the adhesive trap evolving into clam shell-, pitcher- or bladder-like leaves. Furthermore, recent studies have gradually shed light on the molecular mechanisms of trap leaf formation. For *Cephalotus follicularis* or *Utricularia gibba,* the gene expression that controls the adaxial and abaxial domains is suggested to generate leaf specialization (Fukushima *et al.*, 2017; Whitewoods *et al.*, 2020). It has also been further demonstrated that these changes affect growth polarity, which generates cell division orientation and forms leaves with unusual shapes (Whitewoods *et al.*, 2020). The cell division orientation may also be important for shaping the pitcher leaves of *Sarracenia purpurea* (Fukushima *et al.*, 2015).

Elucidation of the structural and functional characteristics of the proteins in the digestive fluid and their genes have greatly advanced in the last two decades. Athauda *et al.* (2004) purified, characterized and sequenced two novel aspartic proteases (nepenthesins I and II) from the digestive fluid of *Nepenthes distillatoria*. Subsequently, a class I chitinase and an S-like ribonuclease (RNase) were found in the digestive fluids of *D. rotundifolia* and *D. adelae*, respectively (Matušíková *et al.*, 2005; Okabe *et al.*, 2005a, b). Class I chitinases break down the exoskeletons of arthropods (mainly insects), releasing the bound nitrogen, and allowing other enzymes to access and degrade internal tissues. Furthermore, these enzymes are also induced in response to pathogen attack or wounding in non-carnivorous plants (Liao *et al.*, 1994; Wu *et al.*, 1994; Hamel and Bellemare, 1995; Gijzen *et al.*, 2001; Zhao *et al.*, 2011). S-like RNases are structurally similar to S-RNases, but functionally different from the S-RNases that function in self-incompatibility (McClure *et al.*, 1989; Huang *et al.*, 1994; McClure *et al.*, 2011). The S-like RNases in non-carnivorous plants respond to the senescence of leaves and flowers, phosphate starvation, pathogen attack and wounding (Nürnberger *et al.*, 1990; Taylor *et al.*, 1993; Bariola *et al.*, 1994; Green, 1994; Ye and Droste, 1996; Galiana *et al.*, 1997; Kariu *et al.*, 1998; Irie, 1999; Hugot *et al.*, 2002).

Okabe and colleagues discovered an S-like RNase in the digestive fluid of *D. adelae*, and hypothesized that carnivorous plants may utilize the defense-related proteins for carnivory (Okabe *et al.*, 2005a). Since then, many such proteins, including proteases, nucleases, chitinases, glucanases, phosphatases, lipases, peroxidases, lipid transfer proteins (LTPs) and thaumatin-like proteins (TLPs), have been identified in the digestive fluids of carnivorous plants (Hatano and Hamada, 2008, 2012; Schulze *et al.*, 2012; Nishimura *et al.*, 2013; Bemm *et al.*, 2016; Lee *et al.*, 2016; Rottloff *et al.*, 2016; Fukushima *et al.*, 2017; Krausko *et al.*, 2017; Matušíková *et al.*, 2018; Kocáb *et al.*, 2020). Amongst them, some had amino acid residues that are conserved only among carnivorous plants (Renner and Specht, 2012; Nishimura *et al.*, 2014; Arai *et al.*, 2015; Fukushima *et al.*, 2017), substantiating the hypothesis proposed by Okabe *et al.* (2005a).

The expression mechanism of the proteins in the digestive fluid differs, depending on the prey-trapping system. In the snap traps, the protein expression is induced. In the pitcher traps and adhesive traps, some enzymes are constitutively expressed, some have induced expression, and others show basically constitutive expression which is occasionally enhanced by the stimulus of trapping prey (Eilenberg and Zilberstein, 2008; Matušíková *et al.*, 2018; Pavlovič and Mithöfer, 2019). Regarding induced expression, mechanical or chemical stimuli induce the expression of the proteins in the digestive fluid (Scala *et al.*, 1969; Matušíková *et al.*, 2005; Eilenberg *et al.*, 2006; Rottloff *et al.*, 2011; Pavlovič *et al.*, 2014), and in some cases jasmonates are involved in this expression (Escalante-Pérez *et al.*, 2011; Libiaková *et al.*, 2014; Bemm *et al.*, 2016; Böhm *et al.*, 2016; Yilamujiang *et al.*, 2016; Krausko *et al.*, 2017; Pavlovič *et al.*, 2017; Pavlovič and Mithöfer, 2019). In non-carnivorous plants, jasmonates regulate the responses to necrotrophic pathogens and herbivore attacks (Zhang *et al.*, 2017), suggesting that carnivorous plants employ the plant defense signaling pathway for inducible expression. On the other hand, the mechanism of constitutive expression has remained poorly understood. The only exception is the study by Nishimura *et al.* (2013) which suggested that the S-like RNase DA-I is constitutively and exclusively expressed in the glandular tentacles of *D. adelae* (Nishimura *et al.*, 2013). Furthermore, the report also proposed that epigenetic regulation is involved in this organ-specific gene expression.

The current study was performed with two purposes: to identify the ‘actors’ that play in the hypothetical epigenetic regulation of *da-I* expression, and to clarify the repertoire, relative amounts and expression mechanisms of other proteins in the digestive fluid of *D. adelae*. We identified and quantitated the proteins in the digestive fluid by a proteomic approach, and examined the relationship between the expression of genes encoding major hydrolytic enzymes and DNA methylation. Our results suggest that *D. adelae* ensures that some of these genes are constitutively expressed via unmethylation, in a glandular tentacle-specific manner, for carnivory.

## Materials and methods

### Plant material

*D. adelae* was purchased from Y’s Exotics (http://ys-exotics.com). The growth conditions have been described previously (Nishimura *et al.*, 2013).

### Database construction for protein identification

The RNA-seq data of *D. adelae* shoots were downloaded from the DNA Data Bank of Japan (DDB) Sequence Read Archive (accession number: DRR051750; Fukushima *et al.*, 2017). After cleaning and quality checks, 31 780 142 confident reads were assembled into 49 512 contigs with an average length of 628 bases, using the de Bruijn graph-based *de novo* assembly program in CLC Genomics Workbench version 8.0.2 (Qiagen, Hilden, Germany) with word size 23, bubble size 50, and minimum contig length 200 bp. Using TransDecoder (Haas *et al.*, 2013), we then identified 20 741 contigs with putative protein-coding regions larger than or equal to 100 amino acids. Among the possible open reading frames (ORFs) in each contig, the most plausible ORF was selected based on the sequence length and two homology searches: a BLASTP search using the UniProt database and an HMMER search using the Pfam-A database for the protein-motifs search, which generated a protein database. Moreover, all protein sequences previously identified in the digestive fluid of *D. adelae* were collected from UniProt and added to our database. To obtain a non-redundant data set, sequences with ≥90% similarity to each other were removed using Cluster Database at High Identity with Tolerance (CD-HIT; Li and Godzik, 2006), which left 19 829 unique contigs in total. The protein sequences in the database were annotated by collating with the *Arabidopsis thaliana* sequences in UniProt/SwissProt, using BLASTP (E-value<10^–4^). The name of the highest scoring protein was used for the annotation.

### Protein digestion

The sticky digestive fluid of *D. adelae* was collected according to the method described by Okabe *et al.* (2005a). The proteins in the digestive fluid were digested with two proteases: Asp-N (Roche Diagnostics, Mannheim, Germany) and chymotrypsin (Promega, Madison, WI, USA). In the Asp-N digestion, a 25 µl solution containing 5 µl digestive fluid, 10 mM DTT, 4.8 M urea and 30 mM Tris-HCl (pH 7.5) was first incubated at 37 °C for 90 min. Iodoacetamide was then added to the solution to a final concentration of 50 mM. After an incubation at ~25 °C for 30 min in the dark, the solution was diluted with 50 mM NH_4_CO_3_ to lower the urea concentration to less than 1 M. Subsequently, Asp-N was added to the solution at a final concentration of 1.2 ng µl^-1^. The solution was incubated at 37 °C overnight. Formic acid was then added to a final concentration of 0.1%, to stop the reaction. The resulting sample was desalted with a GL-Tip SDB (GL Sciences, Tokyo, Japan). After vacuum centrifugation, the peptides were finally dissolved in 50 µl of a solution containing 2% acetonitrile and 0.02% formic acid.

The chymotrypsin digestion was slightly different from the procedure described above. EDTA was added to the solution to a final concentration of 10 mM, and Tris-HCl (pH 8.0) was used in the reduction step with DTT. For dilution, 100 mM Tris-HCl (pH 8.0) was used after the alkylation step with iodoacetamide. In the digestion step, chymotrypsin and CaCl_2_ were added to final concentrations of 12.2 ng µl^-1^ and 10 mM, respectively.

### Sequential window acquisition of all theoretical fragment ion spectra mass spectrometry (SWATH-MS) analysis

The SWATH-MS analysis was performed in two steps. In the first step, ion libraries were generated using information-dependent acquisition (IDA), and in the second, label-free quantification was performed based on the SWATH acquisition. For IDA, the digested peptides were analysed using a Prominence nano system (Shimadzu, Kyoto, Japan) coupled with a TripleTOF 4600 System (AB Sciex, Framingham, MA, USA). Each 4 µl sample was injected onto the trap column (Monolith Trap C18-50–150, 50 µm × 150 mm, Hitachi High-Tech Fielding Corporation, Tokyo, Japan) and desalted with solvent A (2% acetonitrile and 0.1% formic acid in water) at a flow rate of 4 µl min^-1^ for 5 min. Next, the valve position was switched, and the trapped peptides were eluted from a C18 analytical reversed-phased column (MonoCap C18, 50 µm × 150 mm, GL Sciences) at a flow rate of 300 nl min^-1^, with a linear gradient of 2–30% solvent B (98% acetonitrile and 0.1% formic acid in water) over 35 min. The gradient was subsequently increased from 30% to 95% solvent B in 5 min, held for 10 min, and then re-equilibrated with solvent A for 30 min. The valve position was switched back during re-equilibration. The eluate was directed into the nanospray ionization source of the mass spectrometer, with the following source conditions: ion source gas 1, 20 psi; curtain gas, 20 psi; interface heater temperature, 150 °C; ion spray voltage floating, 2300 V. In the MS analysis, we used the positive ion mode over the mass range of m/z 400–1250, with an accumulation time of 250 ms. The 10 most intense precursor ions exceeding 150 counts s^-1^ with charge states of 2–4 were selected for collision-induced dissociation fragmentation, with an ion tolerance of 50 mDa. The dynamic exclusion time was set to 12 s. Tandem mass spectrometry (MS/MS) spectra were acquired over the mass range of m/z 100–1500 with an accumulation time of 100 ms, using the rolling collision energy with a collision energy spread of 15 V.

For the SWATH acquisition, the liquid chromatography (LC) and source conditions were the same as above, and the MS/MS conditions were set using 100 variable windows (https://sciex.com/community/Asset/00001409/vw100_ces_5_10.txt) provided by AB Sciex (Framingham, MA, USA), across the precursor mass range of 400–1250 m z^-1^. A 50 ms survey scan (400–1250 m z^-1^) was acquired at the beginning of each cycle, and MS/MS spectra were collected from 100–1500 m z^-1^ for 25 ms, resulting in a cycle time of 2.7 s.

### Ion library generation

We used the ProteinPilot 5.0 software (AB Sciex) with the Paragon Algorithm (Shilov *et al.*, 2007) to identify the proteins. For each experiment, all IDA data were combined and searched against the putative proteolytic digests of the *D. adelae* proteins described above. The parameters were as follows: sample type, identification; Cys alkylation, iodoacetamide; digestion, Asp-N or chymotrypsin; instrument, TripleTOF 4600; ID focus, biological modifications; search effort, thorough ID. The detected protein threshold was set to 1.3, corresponding to a confidence level of 95%. The resulting files were used as the ion libraries for subsequent SWATH processing.

### Protein quantification

The SWATH data were processed using MS/MS (ALL) with SWATH Acquisition MicroApp 2.0 in PeakView 2.2 (AB Sciex). The parameters were set as follows: 1000 peptides per protein, five transitions per peptide, peptide confidence threshold of 99%, false discovery rate (FDR) threshold of 1%, excluding modified peptides, excluding shared peptides, 5 min of extracted ion chromatogram (XIC) extraction window and 50 ppm of XIC mass tolerance. The retention time was calibrated with endogenous peptides. Based on the resulting data, we excluded reverse sequences and peptides with scores of infinity or FDR ≥1%, and then extracted the intensities of peptides common to two biological replicates. Protein intensities were subsequently calculated by summing all of the peptides for a given protein, and normalized based on the total ion intensity of each sample. Finally, the identified proteins were quantified by the intensity-based absolute quantification (iBAQ) algorithm (Schwanhäusser *et al.*, 2011). The iBAQ values were obtained by the protein intensity divided by the number of peptides theoretically generated by a digestion with Asp-N or chymotrypsin (Supplementary Tables S1; S2).

### Quantification of mRNA

Total RNA was isolated from the glandular tentacles, their heads and stalks, leaves with glandular tentacles removed (hereafter referred to as laminas), roots and inflorescences, using the cetyltrimethyl ammonium bromide-based method (Bekesiova, *et al.*, 1999). The samples were treated with RNase-free DNase I (Promega). First-strand cDNAs were synthesized using ReverTra Ace reverse transcriptase (Toyobo, Osaka, Japan), oligo (dt)_20_ and random primers. Real-time PCR was performed with THUNDERBIRD SYBR qPCR Mix (Toyobo) and specific primers (Supplementary Table S3). Based on the geNorm (Vandesompele *et al.*, 2002) analysis, actin gene, gene encoding eukaryotic initiation factor 4A (*eIF4A*), and gene encoding TIP41 (TAP42 interacting protein of 41 kDa)-like protein (*TIP41*) were selected as the most suitable reference genes. Gene expression was normalized to that of the geometric mean of the reference genes, using the modified Pfaffl method (Zifkin *et al.*, 2012).

### Determination of upstream sequences of genes encoding digestive enzymes

We named the genes encoding the cysteine protease, class I chitinase, β-1, 3-glucanase, TLP and HEL, as *Cysp1*, *Chi1*, *Glu1*, *Tlp1,* and *Hel1*, respectively. Genomic DNA was isolated from leaves, as described above (Bekesiova *et al.*, 1999), and thermal asymmetric interlaced (TAIL)-PCR (Liu *et al.*, 1995; Liu and Whittier, 1995) was performed. The resulting amplified fragments were cloned into the pUC19 vector and sequenced. The putative transcription start sites (TSSs) were determined based on the RNA-seq information (Fukushima *et al.*, 2017), and the *cis*-DNA elements in the region upstream of the TSS were identified by PlantPAN3.0 (Chow *et al.*, 2019).

### Bisulfite sequencing

Genomic DNA samples purified from the glandular tentacles, laminas, roots and inflorescences were treated with sodium bisulfite, using an EpiTect Fast Bisulfite Conversion Kit (Qiagen). Each organ sample was then treated as follows. Using the KOD -Multi & Epi (Toyobo) enzymes and the primers with overhangs for the second PCR (Supplementary Table S4), the following sequences were amplified: *Cysp1*, positions from –336 to +167; *Chi1*, –432 to +36; *Glu1*, –358 to +90; *Tlp1*, –424 to +49; and *Hel1*, –440 to +52. The PCR reactions were repeated three times for each target, and generated 15 full-length replicates in total. After purification, they were combined and the mixture was diluted to 1 ng µl^-1^ with 10 mM Tris-HCl (pH 8.0). Then, using 1 µl of the solution, 10 rounds of PCR amplification were performed with KAPA HiFi HotStart Ready Mix (Roche) and barcode adapters (Fasmac, Kanagawa, Japan). The products were purified with Agencourt AMPure XP beads (Beckman Coulter, Brea, CA, USA) and diluted to 12 ng µl^-1^ with 10 mM Tris-HCl (pH 8.0). Equal amounts of the PCR products of all the organ samples thus obtained were finally combined, and the mixture was sequenced on an Illumina MiSeq (Illumina, San Diego, CA, USA) with 300 bp paired-end reads. The sequencing was performed by Fasmac (Kanagawa, Japan). Using Bisulfighter (Saito *et al.*, 2014), the resulting reads were mapped to the reference sequences, and the percentages of methylated cytosines were determined as follows: the number of cytosines divided by the number of total reads mapped at the same position.

### Identification of cytosine-5 DNA methyltransferase and DNA demethylase

The hidden Markov model profiles of the conserved key domains of cytosine-5 DNA methyltransferase (C5-MTase) (PF00145) and DNA demethylase (PF15628) were downloaded from the Pfam-A database. Using the profiles and HMMER, all possible C5-MTases and DNA demethylases among the *D. adelae* proteins were identified. InterPro was used to confirm and classify each putative C5-MTase or DNA demethylase. In cases where the protein sequences were inadequate for this analysis, we clarified the flanking sequences by inverse PCR or TAIL-PCR. Each protein was named by comparison with the *A. thaliana* sequences in UniProt/SwissProt, using BLASTP (Supplementary Table S5). The protein isoelectric points and molecular weights were determined using ProtParam (https://web.expasy.org/protparam/).

## Results

### Proteins identified in the digestive fluid

**Fig. 1.**
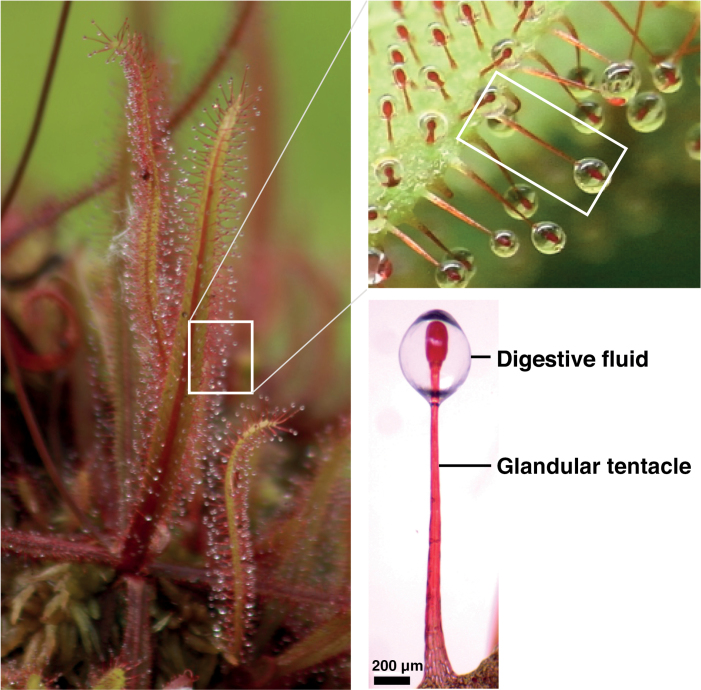
The Australian sundew *D. adelae* and its digestive fluid. The digestive fluid is secreted from the glandular cells forming the tips of the tentacles (scale bar, 200 μm).

In the current study, we did not perform any treatment that could potentially induce gene expression, including prey attachment, and collected the sticky digestive fluid of *D. adelae* (Fig. 1). Using LC-MS/MS-based analysis, we identified 26 proteins in the sticky digestive fluid, of which 19 were novel (Table 1; Supplementary Tables S1; S2; Fig. S1). More than half of the 26 proteins were defense-related proteins, and 11 proteins among them were pathogenesis-related (PR) proteins, which are induced in response to infection by pathogens, including bacteria, fungi, viruses, and viroids (van Loon *et al.*, 2006). Based on iBAQ, the major proteins in the digestive fluid were judged to be TLP, S-like RNase, LTP, cysteine protease, and class I chitinase (Table 1; Supplementary Tables S1; S2). Except for LTP, these proteins are hydrolytic enzymes. S-like RNase and cysteine protease generate ribonucleotides and peptides, respectively. Peptides and ribonucleotides are thought to be used as is, or as the source of amino acids or nitrogen and phosphates, respectively (Nürnberger *et al.*, 1990; Bariola *et al.*, 1994; Okabe *et al.*, 2005a; Godlewski and Adamczyk, 2007; Adamczyk *et al.*, 2009; Nishimura *et al.*, 2014). Chitin is a major structural polysaccharide of arthropods, mollusks and fungi, and class I chitinase hydrolyses the β-1, 4-linkage of chitin, allowing other hydrolytic proteins to penetrate and break down internal tissues. TLP has antifungal activity, and its proposed mechanism of action involves β-1, 3-glucanase activity to disrupt the cell walls of pathogenic fungi (Grenier *et al.*, 1999; Zareie *et al.*, 2002). β-1, 3-glucanase and hevein-like protein (HEL; Brunner *et al.*, 1998), which are considered as hydrolytic enzymes, were also present in the digestive fluid, although they were less abundant than the above-mentioned proteins.

**Table 1.** Proteins in the digestive fluid

Protein name ^a^	Corresponding proteins in *A. thaliana*			Putative physiological function ^c^	Abundance rank ^d^	
	Name ^a^	E-value	Defense-related protein ^b^		Asp-N	Chymotrypsin
Thaumatin-like protein (BCF79772)	Osmotin-like protein OSM34 (P50700)	6.6E-97	● (PR-5)	Antifungal activity	1	1
Unknown (Contig_14 (Fragment))	No Blast hit with E-value <10^–4^	-	-	?	4	2
S-like RNase (BAE16663)	Ribonuclease 1 (P42813)	4.2E-96	●	Prey digestion	3	3
Lipid transfer protein (BAW35429)	No Blast hit with E-value <10^–4^	-	-	?	2	5
Cysteine protease (BAW35427)	Senescence-specific cysteine protease SAG12 (Q9FJ47)	4.5E-108	●	Prey digestion	5	4
Lipid transfer protein (Contig_35636)	Non-specific lipid-transfer protein 11 (Q2V3C1)	4.7E-13	● (PR-14)	Antifungal or antibacterial activity	6	-
Unknown (Contig_1360)	No Blast hit with E-value <10^–4^	-	-	?	7	6
Class I chitinase (BAR13254)	Basic endochitinase B (P19171)	1.5E-151	● (PR-3)	Antifungal activity	8	7
Cysteine-rich repeat secretory protein (Contig_23860)	Cysteine-rich repeat secretory protein 38 (Q9LRJ9)	6.5E-50	?	?	9	8
Lipid transfer protein (Contig_25610 (Fragment))	Non-specific lipid-transfer protein 11 (Q2V3C1)	1.5E-05	● (PR-14)	Antifungal or antibacterial activity	10	-
β-1, 3-glucanase (BAR13253)	Glucan endo-1,3-beta-glucosidase, acidic isoform (P33157)	1.2E-110	● (PR-2)	Antifungal activity	12	-
S1/P1 nuclease (Contig_14977 (Fragment))	Endonuclease 2 (Q9C9G4)	6.7E-51	?	Prey digestion	-	10
Lipid transfer protein (Contig_29120)	Non-specific lipid-transfer protein 4 (Q9LLR6)	5.1E-06	● (PR-14)	Antifungal or antibacterial activity	14	9
Hevein-like protein (BAR13255)	Hevein-like preproprotein (P43082)	1.0E-82	● (PR-4)	Antifungal activity	11	12
GDSL lipase (Contig_30899)	GDSL esterase/lipase APG (Q9LU14)	2.3E-37	?	Prey digestion	15	11
Polyvinylalcohol dehydrogenase (FAA01288)	No Blast hit with E-value <10^–4^	-	-	?	13	13
Lipid transfer protein (Contig_30505)	Non-specific lipid-transfer protein 11 (Q2V3C1)	3.5E-11	● (PR-14)	Antifungal or antibacterial activity	16	-
Polygalacturonase inhibitor (Contig_23225 (Fragment))	Polygalacturonase inhibitor 1 (Q9M5J9)	6.7E-49	●	Antifungal activity	17	-
Valine-tRNA ligase (Contig_1096 (Fragment))	Valine--tRNA ligase, mitochondrial 1 (P93736)	0	?	?	-	14
Class IV chitinase (Contig_23195 (Fragment))	Endochitinase EP3 (Q9M2U5)	1.7E-31	● (PR-3)	Antifungal activity	18	-
S1/P1 nuclease (Contig_3798)	Endonuclease 4 (F4JJL0)	5.2E-138	?	Prey digestion	-	15
Basic secretory protein (BAR13256)	No Blast hit with E-value <10^–4^	-	-	?	19	-
Serine/threonine protein kinase (Contig_9565 (Fragment))	Serine/threonine-protein kinase Nek1 (Q9SLI2)	0	?	?	-	16
Class III peroxidase (Contig_31040 (Fragment))	Peroxidase 51 (Q9SZE7)	4.1E-29	● (PR-9)	Prey digestion	20	-
Calmodulin-binding protein (Contig_21026 (Fragment))	Calmodulin-binding protein 60 B (Q9FKL6)	4.4E-80	?	?	-	17
Class III peroxidase (Contig_46408 (Fragment))	Peroxidase 73 (Q43873)	3.0E-33	● (PR-9)	Prey digestion	21	-

^**a**^ Names in the parentheses indicate the protein ID. The letters starting from ‘Contig’ indicate sequences obtained by *de novo* assembly based on the DRA data DRR051750 (Fukushima *et al.*, 2017), and the others indicate sequences registered with NCBI or UniProt. ^**b**^ ‘PR’ indicates a group of pathogenesis-related proteins. ‘?’ indicates that we could not judge whether the protein was a defense-related protein.  ^**c**^ ‘?’ indicates that function was unpresumable. ^**d**^Table S1 and Table S2 are the bases of ranking. The proteins are listed in descending order based on the normalized average rank of each protein, which was obtained as follows: the abundance rank of each protein in the Asp-N digestion and that in the chymotrypsin digestion were divided by 21 and 17 respectively, and the resulting numbers were averaged (in case where only one digestion hit some protein, its rank in the relevant digestion was used as it was).

### Expression of hydrolytic enzymes

**Fig. 2.**
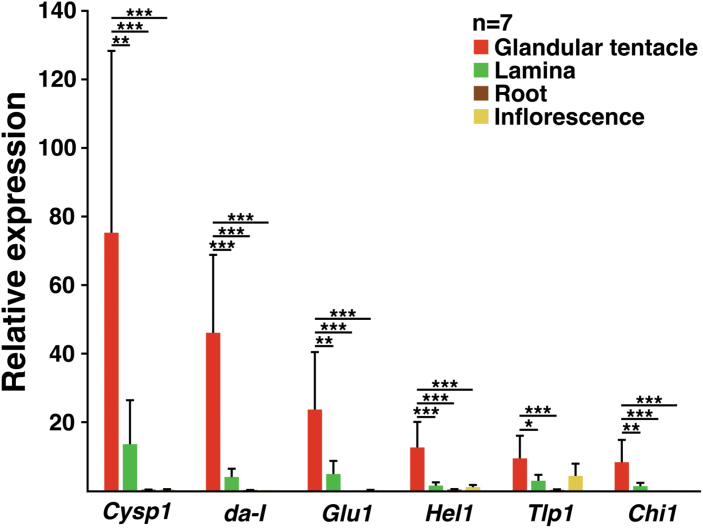
The genes encoding the major hydrolytic enzymes in the digestive fluid are expressed in a glandular tentacle-specific manner. The expression of *Cysp1, da-I*, *Glu1, Hel1, Tlp1,* and *Chi1* in glandular tentacles, laminas, roots and inflorescences were examined and normalized to that of the geometric mean of the reference genes (*actin, eIF4A, TIP41*). The values are presented as the mean ±SD (*n*=7 biological replicates). *P* values were calculated by Tukey’s HSD test (*P<0.05; **P<0.01, ***P<0.001).

We examined the expression of genes encoding the six hydrolytic enzymes in the digestive fluid. For this experiment, *D. adelae* was dissected into glandular tentacles, laminas, roots and inflorescence, as described previously (Nishimura *et al.*, 2013). All genes encoding hydrolytic enzyme were almost exclusively expressed in the trap organ (glandular tentacles), at considerably higher amounts than that of the geometric mean of the reference genes (*actin, eIF4A, TIP41*; Fig. 2). We previously reported the glandular tentacle-specific expression of *da-I* (Nishimura *et al.*, 2013). However, the fold-expression of this gene was much higher in the present analysis. This may be related to the ‘seasonal vital difference’ of the plant: in the current study, glandular tentacles were dissected in June (rainy season in Japan), while in the previous study (Nishimura *et al.*, 2013), they were dissected at the end of August. The former plants were more healthy than the latter, under our growth conditions (Nishimura *et al.*, 2013). The high expression of *Cysp1* and *da-I* in the glandular tentacles correlated with their corresponding protein contents, which were quite high (Table 1).

**Fig. 3.**
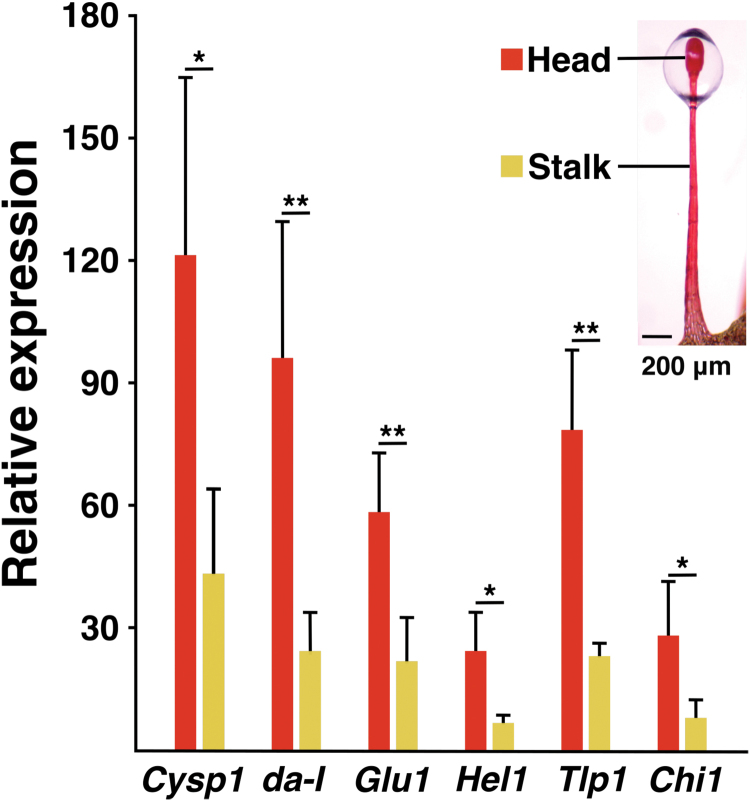
Expression of *Cysp1, da-I*, *Glu1, Hel1, Tlp1* and *Chi1* in the head and stalk parts of glandular tentacles. Gene expression was normalized to that of the geometric mean of the reference genes (*actin, eIF4A, TIP41*). The values are presented as the mean ±SD (*n*=4 biological replicates). *P* values were calculated by Student’s *t*-test. (*P<0.05; **P<0.01).

The glandular tentacles can be divided into two parts: head and stalk (Lloyd, 1942; Williams and Pickard, 1974; Outenreath and Dauwalder, 1982; Naidoo and Heneidak, 2013). Thus, we also examined the expression of *Cysp1*, *da-I, Chi1*, *Glu1*, *Tlp1,* and *Hel1* in each part (Fig. 3), and found that most of the transcripts collected from the glandular tentacles were derived from the heads. In *Drosera capensis*, the head is mostly composed of the outer digestive gland cells (ODGC) and the inner digestive gland cells (IDGC; Williams and Pickard, 1974; Outenreath and Dauwalder, 1982). Given the similarity between *D. capensis* and *D. adelae* (Cameron *et al.*, 2002), the six genes of interest were also considered to be transcribed in these cells in *D. adelae*. Lower amounts of these gene transcripts were also found in the stalks (Fig. 3). The stalk mostly consists of outer and inner stalk cells, and it also has sessile gland cells (SGC). Most of all of these cells may be implicated in this phenomenon.

### DNA methylation profiles of genes encoding hydrolytic enzymes

In eukaryotes, DNA methylation is a chemical modification of genomes that affects gene expression and genome stability (Law and Jacobsen, 2010; Zhang *et al.*, 2018). DNA methylation in plants commonly occurs in three cytosine sequence contexts: CG, CHG and CHH (where characters C and G represent nucleotides C and G, and H means non-G nucleotides; Zhang *et al.*, 2006; Cokus *et al.*, 2008; Lister *et al.*, 2008). We previously reported that *da-I*, which is highly and specifically expressed in glandular tentacles, is only unmethylated in that organ, and highly methylated in other organs (Nishimura *et al.*, 2013). This suggested that DNA methylation controls the expression of *da-I*. To determine whether the same phenomenon is observed in the hydrolytic enzyme genes described above, we analysed the DNA methylation profiles of the regions upstream of their TSSs.

**Fig. 4.**
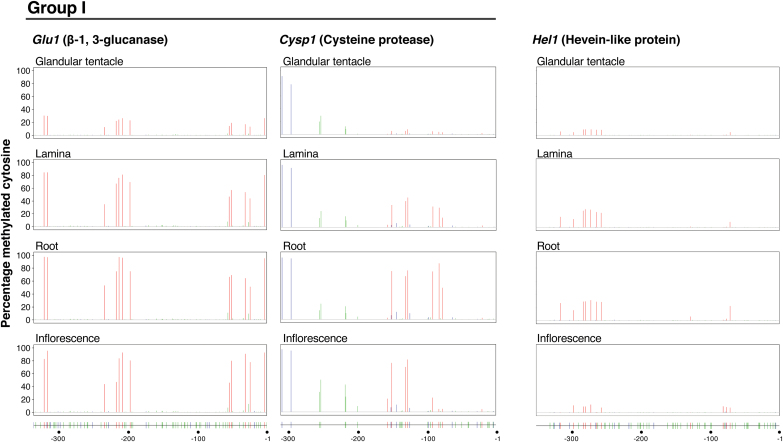

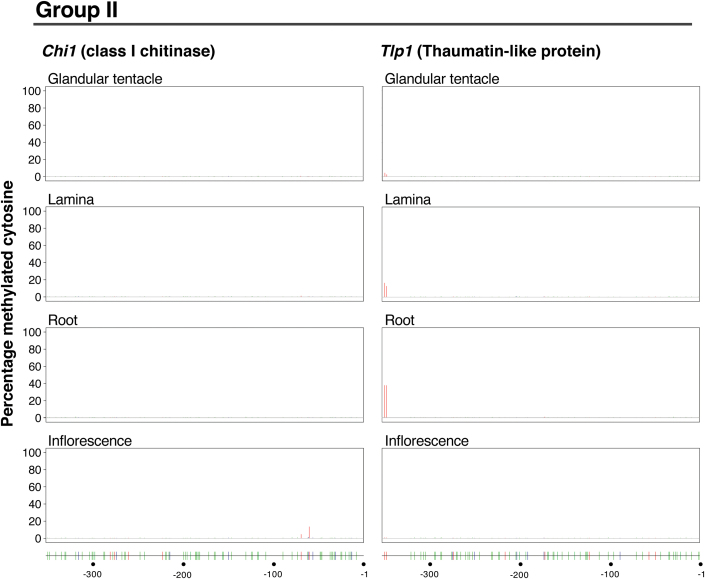
Organ-dependent DNA methylation profiles of the genes encoding the major hydrolytic enzymes. Focusing on the genes *Glu1*, *Cysp1, Hel1*, *Chi1* and *Tlp1*, the DNA methylation status of the upstream region of the TSS was examined in several organs, including glandular tentacles, laminas, roots and inflorescences. Histograms show the percentages of methylation at cytosine residues in CG, CHG and CHH contexts: CG, red; CHG, blue; CHH, green (averages of three individual plants). The lower diagrams show the positions of cytosines in the same contexts with the same colors. According to the methylation characteristics, the genes were grouped as group I, almost unmethylated only in glandular tentacles; group II, almost unmethylated in all organs examined.

The DNA methylation profiles for *Glu1* and *Cysp1* (Fig.4) were somewhat similar to that of *da-I* (Nishimura *et al.*, 2013). On the other hand, the profiles for *Chi1* and *Tlp1* considerably differed from the *da-I* profile: for these genes, methylation was generally not found or quite restricted. We named *da-I*, *Glu1* and *Cysp1* as the group I genes, and *Chi1* and *Tlp1* as the group II genes. The *Hel1* profile appeared to be in between those of the two groups. The methylation profile of *Glu1* was the closest to that of *da-I* (Nishimura *et al.*, 2013), in the sense that the extent of methylation at the CG sites was generally high except for glandular tentacles and that the methylation at CHG and CHH sites was generally low in all organs examined (these sites were almost unmethylated in *Glu1*), although a small population of cells had methylated CG sites in the glandular tentacles in the case of *Glu1* (Fig. 4). The differentially methylated regions (DMRs) for the CG sites of *Glu1* were −322 to −317, −235 to −198 and −55 to −4, and the percentages of the methylation in this order were 30%, 21%, and 18% in glandular tentacles, 84%, 66%, and 56% in laminas, 97%, 79%, and 69% in roots, and 89%, 69%, and 77% in inflorescences, respectively. The CG sites in *Cysp1* were almost unmethylated in the glandular tentacles, and were highly, regionally high, or moderately methylated in roots, inflorescences and laminas, respectively. For the methylations at the CHG and CHH sites in the gene, the profiles were almost the same amongst the organs, and the methylation extents were very low in the region from ~ −200 to −1. The DMRs for the CG sites of *Cysp1* were from positions −157 to −128 and −92 to −78, and the percentages in this order were 5% and 4% in glandular tentacles, 31% and 26% in laminas, 55% and 70% in roots, and 62% and 10% in inflorescences, respectively. Regarding *Hel1*, the population of cells with methylated CG sites was small, and methylated CHG and CHH sites were not detected in all organs. For *Hel1*, the position of the DMR was from −317 to −258 and the percentages were 8% in glandular tentacles, 22% in laminas, 27% in roots, and 7% in inflorescences.

### Organ-specific expression of cytosine-5 DNA methyltransferases and DNA demethylases

Plant C5-MTases can be divided into three families: methyltransferase (MET) family, chromomethylase (CMT) family, and domains rearranged methyltransferase (DRM) family (Finnegan *et al.*, 1996; Lindroth *et al.*, 2001; Cao and Jacobsen, 2002; Stroud *et al.*, 2014). Plants also have a DNA demethylase family. In *A. thaliana*, this family comprises four members: REPRESSOR OF SILENCING 1 (ROS1), DEMETER (DME), DEMETER-LIKE PROTEIN 2 (DML2) and DML3 (Choi *et al.*, 2002; Gong *et al.*, 2002; Ortega-Galisteo *et al.*, 2008), which can excise the methyl group of 5-^me^C from all cytosine sequence contexts (Agius *et al.*, 2006; Gehring *et al.*, 2006; Morales-Ruiz *et al.*, 2006; Penterman *et al.*, 2007; Zhu *et al.*, 2007). We then analysed the expression of the C5-MTases and DNA demethylases in the relevant organs. To obtain the sequence information of these enzymes, the *D. adelae* proteins were screened using HMMER, which allowed us to identify the putative complete or partial coding sequences of three C5-MTase and two DNA demethylase genes (Supplementary Table S5).

The three C5-MTases were named DaMET1, DaCMT3, and DaDRM2 (Supplementary Table S5). DaMET1 was confirmed to have two replication foci domains, two bromo adjacent homology (BAH) domains and a C-terminal DNA MTase domain, usually found in the MET family. This family is used for maintenance methylation and methylates CG (Finnegan *et al.*, 1996; Kankel *et al.*, 2003). DaCMT3 was also confirmed to have a BAH domain and a chromodomain within the C-terminal DNA MTase domain, as commonly found among the CMT family enzymes. The CMT3 binds to H3K9me2 with its BAH and chromodomain, and maintains CHG methylation (Du *et al.*, 2012). Regarding DaDRM2, we found two ubiquitin-associated domains in its N-terminal region and a DNA MTase domain in its C-terminal region, and thus confirmed that it belongs to the DRM family. This family is used for both the *de novo* methylation in all sequence contexts (CG, CHG and CHH) and the maintenance of CHH methylation, through the RNA-directed DNA methylation pathway (Matzke and Mosher, 2014). Although the sequence of DaCMT3 was partial, it was used in subsequent analysis because it contained all of the characteristic domains of CMT.

Each of the two putative DNA demethylases identified in the current study contains a helix-hairpin-helix Gly/Pro/Asp domain, a permuted single zinc finger-CXXC unit domain and an RNA recognition motif domain (Law and Jacobsen, 2010), which are also present in most plant DNA demethylases. Furthermore, the search against the *A. thaliana* protein database (UniProtKB/Swiss-Prot) revealed that one was most similar to DME, and the other was similar to ROS1. Thus, they were named DaDME and DaROS1, respectively (Supplementary Table S5).

**Fig. 5.**
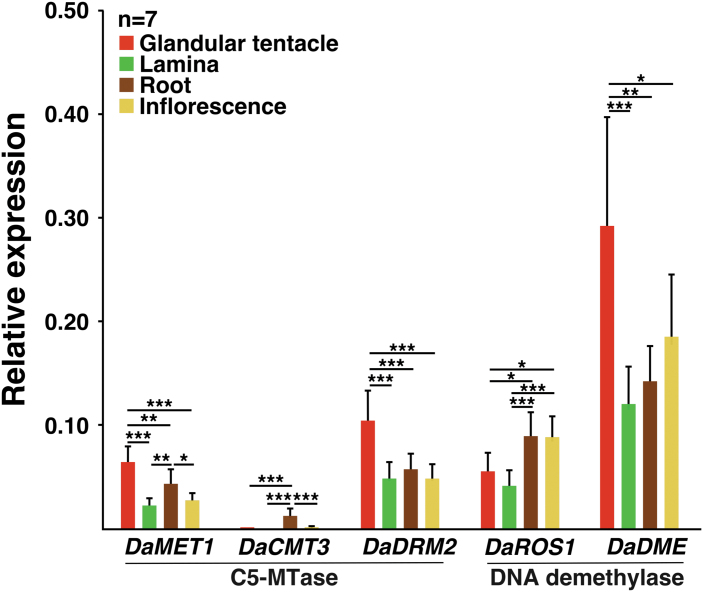
Expression of the genes encoding C5-MTase and DNA demethylase. Using glandular tentacles, laminas, roots and inflorescences, the expression of the relevant genes was examined and normalized to that of the geometric mean of the reference genes (*actin, eIF4A, TIP41*). The values are represented as the mean ±SD (*n*=7 biological replicates). *P* values were calculated by Tukey’s HSD test (*P<0.05; **P<0.01; ***P<0.001).

The relative expression of the identified genes (*DaMET1*, *DaCMT3*, *DaDRM2*, *DaDME* and *DaROS1*) in glandular tentacles, laminas, roots and inflorescences were analyzed by qPCR (Fig. 5). The expression of three genes *DaMET1*, *DaDRM2* and *DaDME* were higher in the glandular tentacles than in the other organs. Notably, in the glandular tentacles, the expression of *DaDME* was considerably higher than those of the other four genes. *DaCMT3* expression was almost undetectable in all organs except roots.

## Discussion

### Similarity of proteins in the digestive fluids of *D. adelae* and other carnivorous plants

Many of the major proteins in the digestive fluid of *D. adelae* (Table 1) are also present in other carnivorous plants; e.g. *D. muscipula*: an S-like RNase, cysteine proteases, a class I chitinase, a β-1, 3-glucanase(s), a TLP, LTPs and a HEL (Schulze *et al.*, 2012; Nishimura *et al.*, 2013; Bemm *et al.*, 2016); *Nepenthes alata*: an S-like RNase, β-1, 3-glucanases, a TLP(s) and LTPs (Hatano and Hamada, 2008, 2012; Rottloff *et al.*, 2016; Fukushima *et al.*, 2017); *C. follicularis*: an S-like RNase, a β-1, 3-glucanase and TLPs (Nishimura *et al.*, 2013; Fukushima *et al.*, 2017); and *S. purpurea*: β-1, 3-glucanases and a TLP (Fukushima *et al.*, 2017). The major proteins in the digestive fluid of *D. muscipula* are very similar to those of *D. adelae* (Schulze *et al.*, 2012). A β-1, 3-glucanase and TLP are also predominant in the digestive fluid of *N. alata* (Hatano and Hamada, 2008). Importantly, many of these proteins also function in the roots of non-carnivorous plants (Nóbrega *et al.*, 2005; Basu *et al.*, 2006; De-la-Peña *et al.*, 2008, 2010; Shinano *et al.*, 2011, 2013).

The protease in the digestive fluid of *D. adelae* belongs to the cysteine protease family (Table 1). Similarly, *D. muscipula* predominantly secretes cysteine proteases in the digestive fluid (Schulze *et al.*, 2012; Bemm *et al.*, 2016). On the other hand, *Nepenthes* and *C. follicularis* mainly secrete aspartic proteases (Hatano and Hamada, 2008, 2012; Rottloff *et al.*, 2016; Fukushima *et al.*, 2017), similar to the *D. capensis* digestive fluid that has an aspartic protease (Takahashi *et al.*, 2009; Krausko *et al.*, 2017). At present, we do not understand the reason for these differences. There are two possibilities to consider whether *D. adelae* actually has an aspartic protease in its digestive fluid. Firstly, *D. adelae* could indeed lack aspartic proteases in its digestive fluid; secondly, the proteomic analysis was not exhaustive and the aspartic protease could remain undetected. We expect the former possibility to be more likely, for the following reason. Although the database constructed for protein identification might not be exhaustive, it contained the sequences of 13 putative aspartic proteases. If an aspartic protease was present in the digestive fluid of *D. adelae*, then there is a strong possibility that it would have been detected by the analysis. However, we only detected the cysteine protease CYSP1. It must also be noted that the constructed database was based on the transcriptome data of shoots (Fukushima *et al.*, 2017). Thus, it seems that the expression of the aspartic proteases is either very low or does not occur in the glandular tentacles of *D. adelae*.

### Similarity between trap organs and roots as deduced by the proteins in the digestive fluids

**Fig. 6.**
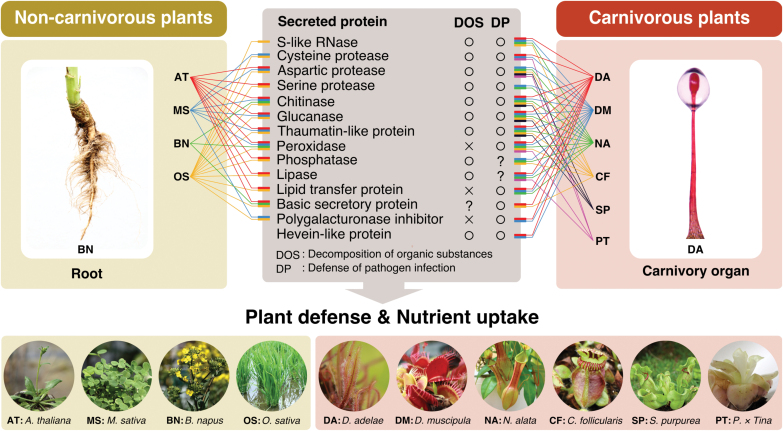
Similarity between the proteins in the digestive fluids of carnivorous plants and those secreted from the roots of non-carnivorous plants. The relevant proteins are aspartic protease, basic secretory protein, chitinase, cysteine protease, glucanase, hevein-like protein, lipase, lipid transfer protein, peroxidase, phosphatase, polygalacturonase inhibitor, S-like RNase, serine protease and thaumatin-like protein (shown in the center panel). Many or some of them were found in the digestive fluids of *D. adelae* (abbreviated as DA), *D. muscipula* (DM), *N. alata* (NA), *C. follicularis* (CF), *S. purpurea* (SP) and *Pinguicula* × *Tina* (PT) (right panel), and also found to be secreted from the roots of *A. thaliana* (AT), *M. sativa* (MS), *B. napus* (BN) and *O. sativa* (OS) (left panel). DOS and DP stand for ‘decomposition of organic substances’ and ‘defense of pathogen infection’, respectively. The data sources were as follows: *A. thaliana*, Basu *et al.*, 2006, De-la-Peña *et al.*, 2008, 2010, Tran *et al.*, 2010; *M. sativa*, De-la-Peña *et al.*, 2008; *B. napus*, Basu *et al.*, 2006; *O. sativa*, Shinano *et al.*, 2011, 2013; *D. muscipula*, Schulze *et al.*, 2012, Bemm *et al.*, 2016; *N. alata*, Hatano and Hamada, 2008, 2012, Rottloff *et al.*, 2016, Fukushima *et al.*, 2017; *C. follicularis*, Fukushima *et al.*, 2017; *S. purpurea*, Fukushima *et al.*, 2017; *P.* × *Tina*, Kocáb *et al.*, 2020.

The carnivory organ (glandular tentacles) of *D. adelae* is similar to roots in terms of function, i.e. both organs can incorporate nutrients, protect themselves against pathogen infection, and establish symbiotic relationships with microorganisms. As for nutrient incorporation from prey, the S-like RNase DA-I and the cysteine protease CYSP1, which are highly abundant in the digestive fluid (Table 1), are thought to play the central role in *D. adelae*. In non-carnivorous plants, nucleases and proteases are considered to be secreted from roots upon phosphate or nitrogen deficiency, thereby allowing plants to efficiently absorb the nutrients present in soil (Chen *et al.*, 2000; Godlewski and Adamczyk, 2007; Paungfoo-Lonhienne *et al.*, 2008; Adamczyk *et al.*, 2008, 2009; Adamczyk, 2014). Except for these enzymes, many of the proteins in the digestive fluid were ‘defense-related proteins’, of which most were PR proteins (Table 1). For example, chitinase can decompose the cell walls of fungi (Schlumbaum *et al.*, 1986; Mauch *et al.*, 1988; Sela-Buurlage *et al.*, 1993), and TLP has antifungal activity (Grenier *et al.*, 1999; Zareie *et al.*, 2002). Thus, these proteins are thought to be used in a defense system against fungal attacks. Similarly, the major proteins in the digestive fluids of *D. muscipula, N. alata, C. follicularis* and *S. purpurea* were also defense-related proteins (Hatano and Hamada, 2008, 2012; Schulze *et al.*, 2012; Nishimura *et al.*, 2013; Bemm *et al.*, 2016; Rottloff *et al.*, 2016; Fukushima *et al.*, 2017), although their expression in *D. muscipula* is inductive. In non-carnivorous plants, including *A. thaliana*, *Medicago sativa*, *Brassica napus* and *Oryza sativa*, many of the defense-related proteins shown in Fig. 6 are constitutively or inducibly secreted from the roots to the rhizosphere (Nóbrega *et al.*, 2005; Basu *et al.*, 2006; De-la-Peña *et al.*, 2008, 2010; Shinano *et al.*, 2011, 2013). Thus, there is close similarity between the digestive fluid proteins of carnivorous plants and those secreted from the roots of non-carnivorous plants (Fig. 6). The S-like RNases and proteases also play an important role in the self-defense system, which functions upon wounding and pathogen attack (Ye and Droste, 1996; Galiana *et al.*, 1997; Kariu *et al.*, 1998; Hugot *et al.*, 2002; Hou *et al.*, 2018). We did not examine the symbiotic relationship of carnivorous plants with microorganisms. However, carnivory organs generally harbor symbiotic bacteria and fungi that facilitate nutrient incorporation (Albino *et al.*, 2006; Cao *et al.*, 2015; Chan *et al.*, 2016; Bittleston *et al.*, 2018; Sirová *et al.*, 2018). Similarly, the roots of non-carnivorous plants generally receive benefits from these microorganisms (Lugtenberg and Kamilova, 2009; Berendsen *et al.*, 2012; Martin *et al.*, 2017).

In addition to these comparable features, recent studies have revealed another similarity between carnivory organs and roots. Transporters are involved in nutrient absorption via roots. The same transporters are used in the carnivory organs of some carnivorous plants, presumably to absorb nutrients from prey: e.g. *N. alata*, *D. muscipula* and *C. follicularis* use ammonium transporter 1 (AMT1; Schulze *et al.*, 1999; Scherzer *et al.*, 2013; Fukushima *et al.*, 2017), and *D. muscipula* uses phosphate transporter 1 (PHT1; Bemm *et al.*, 2016). Moreover, transcriptome analyses of various *D. muscipula* tissues demonstrated closest similarity between its glandular tissues and roots, for the genes implicated in protein metabolism, transport and stress responses (Bemm *et al.*, 2016; Palfalvi *et al.*, 2020). Thus, carnivorous plants may have generally acquired the ability to absorb nutrients from their prey via leaves as an auxiliary mechanism for adaptation to nutrient- poor habitats.

### Expression of genes encoding hydrolytic enzymes

The hydrolytic enzyme-encoding genes *Cysp1, Chi1, Glu1, Tlp1* and *Hel1* were almost exclusively expressed in glandular tentacles (Fig. 2). Furthermore, the heads generated the greatest proportion of the transcripts (Fig. 3). According to the studies using *D. capensis*, the head of the glandular tentacle contains ODGC, IDGC, endodermal cells forming a continuous layer that separate the digestive gland cells from the central conducting tissues, tracheids, and neck cells (Williams and Pickard, 1974; Outenreath and Dauwalder, 1982). Amongst these, ODGC and IDGC form the majority of the cell population. On the other hand, the stalk mostly consists of outer and inner stalk cells, and it also has SGC (Lloyd, 1942; Williams and Pickard, 1974; Naidoo and Heneidak, 2013). The cell composition in the glandular tentacles seems similar between *D. adelae* and *D. capensis* (Cameron *et al.*, 2002). Accordingly, for heads, the major population of the transcripts of the six genes shown in Fig. 2 were most likely derived from ODGC and IDGC. For stalks, these genes may be expressed in most or all of the cells described above. For the inner stalk cells, a study using *D. rotundifolia* previously reported that induction-independent chitinase transcripts are confined to the cells (Matušíková *et al.*, 2005). The study using *D. capensis* showed that SGC also has a secretory function (Naidoo and Heneidak, 2013). The secreted fluid from the SGC may be transported into the head area.

DNA methylation profiles of the group I genes (*da-I*, *Glu1* and *Cysp1*) and those of the group II genes (*Chi1* and *Tlp1*) were distinct (for *da-I*, Nishimura *et al.*, 2013; for the other genes, Fig. 4). The CG sites of *Glu1* and *Cysp1* were almost unmethylated in the upstream region of each TSS in glandular tentacles, but highly methylated in laminas, roots and inflorescences (Fig. 4). These profiles were very similar to those of *da-I* (Nishimura *et al.*, 2013). Therefore, the glandular tentacle-specific expression of these genes may also be explained in terms of epigenetic regulation, based on open and closed chromatin structures, as previously hypothesized for *da-I* expression by Nishimura *et al.* (2013). A transcription factor-implicated mechanism is also possible, as some transcription factors cannot bind to their target sequences when they are methylated, thus preventing transcription initiation (Mann *et al.*, 2013; O’Malley *et al.*, 2016; Yin *et al.*, 2017). If the latter mechanism actually exists, then some DNA motifs that act in *cis* should be identified in the DMRs: *da-I*, –349 to –307, –257 to –205, –139 to –97 and –50 to –24 (Nishimura *et al.*, 2013); *Glu1*, −322 to −317, −235 to −198 and −55 to −4; and *Cysp1*, −157 to −128 and −92 to −78. However, we could not detect any known *cis* DNA element or *trans*-acting factors that are likely to be responsible for this hypothetical mechanism (Okabe *et al.*, 2005b; Supplementary Fig. S2).

On the other hand, the upstream regions of the TSSs of *Chi1* and *Tlp1* (group II) were almost completely unmethylated in all organs (Fig. 4). However, the expression of these genes was glandular tentacle-specific (Fig. 2). These observations strongly suggest that glandular cells contain some key, but unknown transcription factor(s) that can drive the transcription of these genes. Notably, the genes encoding the S-like RNases *dm-I* of *D. muscipula* and *cf-I* of *C. follicularis* have characteristics similar to those of the group II genes (Nishimura *et al.*, 2013). Thus, the orthologs are not necessarily regulated in the same way. In the case of *Hel1*, its methylation profiles were in between those of groups I and II (Fig. 4). Considering its extent of methylation, however, DNA methylation may be irrelevant to its expression. We found a PHR1 (phosphate starvation response 1)-binding sequence (P1BS), a phosphate starvation responsive sequence (Rubio *et al.*, 2001), in the region spanning from −317 to −258 (Fig. 4; Supplementary Fig. S2). Phosphate starvation may activate PHR1, which could then bind to P1BS and trigger *Hel1* expression in glandular tentacles. The P1BS motif is also present in the *da-I* promoter (Okabe *et al.*, 2005b) and the promoter of the *RNase T2* gene that shows trap-specific expression in *U. gibba* (Oropeza-Aburto *et al*., 2020) which seems very intriguing.

### Implications of the glandular tentacle-specific DNA demethylation

The methylation profiles of the group I genes (Fig. 4; Nishimura *et al.*, 2013) suggested two possibilities: demethylation of these genes occurs in a glandular tentacle-specific manner, or their methylation specifically occurs in laminas, roots and inflorescences. The expression of *DaMET1* and *DaDRM2* was very low in laminas, roots and inflorescences, while in the glandular tentacles, *DaDME* expression was considerably higher than those of the other genes (Fig. 5). DaMET1 and DaDRM2 are MTases and DaDME is a demethylase. Thus, the data shown in Fig. 5 support the possibility that the demethylation of the group I genes (*da-I, Glu1* and *Cysp1*) occurs in a glandular tentacle-specific manner. To our knowledge, tissue-specific DNA demethylation by DME confers distinct gene expression during gamete formation and nodule development in non-carnivorous plants (Gehring *et al.*, 2009; Hsieh *et al.*, 2009; Ibarra *et al.*, 2012; Satgé *et al.*, 2016). Thus, we may consider the glandular tentacle-specific demethylation of the group I genes as a similar type of gene regulation, in the sense that organ-specific DNA demethylation by DME presumably occurs in the differentiation process of glandular tentacles. Clearly, follow on work should examine the timing of promoter methylation or demethylation in the process of *D. adelae* organogenesis.

### Conclusions

Our results substantiate the following hypothesis, in which a part was previously hypothesized regarding the abundant presence of DA-I in the digestive fluid (Okabe *et al.*, 2005a). The ancestors of *D. adelae* must have engendered the development of carnivory in their leaves to adapt to nutrient-deficient habitats and, at the same time, establish self-defense mechanisms against pathogen attack and mechanical injury caused by insects. Furthermore, these features could be attained by slight modifications of the expression mechanisms of a set of genes that are generally used for specific functions of roots, including the epigenetic regulation shown in this study. A somewhat similar phenomenon occurs in the roots of some non-carnivorous plants: phosphate or nitrogen starvation causes the plants to express genes for nutrient absorption in the roots by altering DNA methylation patterns (Secco *et al.*, 2015; Yong-Villalobos *et al.*, 2015; Mager and Ludewig, 2018). To understand the evolution of carnivorous plants, it seems absolutely necessary to further explore similarities between roots of non-carnivorous plants and trap-organs of carnivorous plants, such as in gene usage and regulatory mechanisms of gene expression.

## Supplementary data

The following supplementary data are available at *JXB* online.

Fig. S1. FASTA file of amino acid sequences of the proteins subjected to LC-MS/MS analysis

Fig. S2. Upstream sequences and *cis*-DNA elements of *Cysp1*, *Chi1*, *Glu1*, *Tlp1* and *Hel1*

Table S1. Summary of iBAQ values in the Asp-N digestion

Table S2. Summary of iBAQ values in the chymotrypsin digestion

Table S3. PCR primers used in the Real-Time PCR

Table S4. PCR primers used in the bisulfite sequencing

Table S5. C5-MTases and DNA demethylases identified in *D. adelae*

eraa560_suppl_Supplementary_Figures-S1-S2_and_Tables-S1-S5

## Data availability

The mass spectrometry proteomics data have been deposited to the Proteome Xchange Consortium *via* the jPOST partner repository (Okuda *et al.*, 2017) with the dataset identifier PXD022147. The bisulfite sequence data have been deposited to DDBJ (accession number:DRA010963). Sequence data can be found in the DDBJ data libraries under the accession numbers LC037411 (*Bsep1*), LC037409 (*Chi1*), LC549487 (*Cysp1*), LC037408 (*Glu1*), LC037410 (*Hel1*), LC547507 (*Tlp1*), BR001645 (*polyvinylalcohol dehydrogenase* gene), YAAA01000001 (*DaMET1*), YAAA01000002 (*DaCMT3*), YAAA01000003 (*DaDRM2*), YAAA01000004 (*DaROS1*), YAAA01000005 (*DaDME*), LC589199 (*eIF4A*) and LC589200 (*TIP41*).

## Abbreviations

AMT1: ammonium transporter 1
BAH: bromo adjacent homology
C5-MTase: cytosine-5 DNA methyltransferase
CMT: chromomethylase
DME: DEMETER
DML: DEMETER-LIKE PROTEIN
DMR: differentially methylated region
DRM: domains rearranged methyltransferase
FDR: false discovery rate
HEL: hevein-like protein
iBAQ: intensity-based absolute quantification
IDA: information-dependent acquisition
IDGC: inner digestive-gland cells
LTP: lipid transfer protein
MET: methyltransferase
ODGC: outer digestive-gland cells
P1BS, PHR1: -binding sequence
PHR1: phosphate starvation response 1
PR: pathogenesis-related
RNase: ribonuclease
ROS1: REPRESSOR OF SILENCING 1
SGC: sessile gland cells
SWATH-MS: sequential window acquisition of all theoretical fragment ion spectra mass spectrometry
TAIL: thermal asymmetric interlaced
TLP: thaumatin-like protein
TSS: transcription start site
XIC: extracted ion chromatogram.
