# Supplementary material for: Organ-specific expression and epigenetic traits of genes encoding digestive enzymes in the lance-leaf sundew (Drosera adelae)
Source: J Exp Bot. 2020 Nov 28;72(5):1946–61. doi: 10.1093/jxb/eraa560 (PMC7921302; doi:10.1093/jxb/eraa560)
Supplement: eraa560_suppl_Supplementary_Figures-S1-S2_and_Tables-S1-S5 [file eraa560_suppl_supplementary_figures-s1-s2_and_tables-s1-s5.pdf]

>Thaumatin-like protein (BCF79772)

MSNLIISFFLFSILFSVSTNAATFTITNNCPYTIWAAAVPGGGKQLNSGGTWTITAPGGTTGARIWGRTGCTVT
GPNGLRCNTGDCGGLFQCQGYGQPPNTLAEYALNQFDNLDFFDISLVDGFNVPMQFVPVSGGCTSGPICSANII
AQCPSVLQTQGGCNNPCTVFKTPEYCCTSGSCGPTQYSEFFKGLCPNAYSYPKDDATSTYTCKTGANYKVVFCP

>Unknown (Contig_14 (Fragment))

MAMASPSLHKIVFACGAITIMLMAIGAIIPSTDAAMFIFTPNPLYYCRDCIANGAKASLLSWGTCCPGLCDLAK
TLATSKARLVDKQLGCLGMSQYLSEIPGYSYV

>S-like RNase (BAE16663)

MENYSMIIKLLVWQSLAAMALCQSPGFDFFYFVQQWPGAYCDTSRGCCNPTSGKPPSDFGIHGLWPNYNSGGYP
SNCDSSNPFDPSQIQDLLSQLQTQWPSLTCPSSDGTSFWTHEWNKHGTCSESVLTEHAYFAAALNLKSQANTLA
SLTNAGITPNNSFYNLSDVLAAIKQGTGHDAYVQCNTDENGNSQLYQIYICVDTTGANFIECPVAPNQNCPSSI
EFPSF

>Lipid transfer protein (BAW35429)

MAAGLKLATLAIWSCLVIVGVAPQASYAAFIGYDWGTTFFYCCKDYIQTGIDCALDYACCPVIAQFAGAVNKAS
TKDEISYSCLAMKSALYSMPYYNYTATATMVAKCGYTLPYNVAKTAPCY

>Cysteine protease (BAW35427)

MAFTNTFHQFLLFAILLCIGLSSFQSSAISHEPSMVKRHEEWMVQHRRTYKDNVEKERRFQIFQKNVNLIEAHN
KKNKSYSLSVNQFADLTDEEFEKMYASAKVPESRHSNSQRTPFKYEGYTSVPASINWVTMGAVTPIRNQGTCGS
CWAFAAIATVESLTWIKTGKSYDLSEQQIVDCDRYGKDRGCNGGYADGAYNWIINNRGVTTEASYPYVGYQQYY
CYRAQSAVTIKGYQFVPNNSERAMQQAVANQPIAVYVESKGYNFKYYSGGLFTGQCGTATDHVVAVVGYGTTTD
GYPYWLIKNSWGTGWGENGYMRMLRNVNDPRGLCGIALYPTYPTN

>Lipid transfer protein (Contig_35636)

MAGFSVKFSAALLISILLVAGIASFTAEASVCGQVAVVSRECMPYVMDKAPSVTSSCCSATVGLASLLNTAANK
KQACQCLKDIASKMPGIDYNRVKSIPSKCGIKHFPLPLDPNAKC

>Unknown (Contig_1360)

MAMATSRSVLHLKFACIAALVVILAMNPAHFVDAGIFTFTDNCLYYCQDCLAHGAIASVFSWLGCCPGMQRDAK
KLNTPGCDMRDKAFACTGYKAYLMDFSGYNYDNTQYTLKRCNASFPFPVSTSVDCFKAVS

>Class I chitinase (BAR13254)

MKISLLLLLFVVPLLSGTSAQQCGYQAGGALCPNGLCCSKYGYCGTTSAYCGDGCQSQCGGSSPPPAPPSPPSP
SGGGDVSSIITSQIFNQMLLHRNDNACPANGFYSYQAFINAARKFSGFGTTGDTNTRKNELAAFFGQTSHETTG
GWSTAPDGPYAWGYCFKQEQGNPGDYCAPSSTYPCAPGQKYYGRGPIQISYNYNYGLCGAAINQPLLSNPGLVA
SDADISFETAIWFWMTPQGNKPSCHAVATGQWNPSSADQAAGRVPGYGVITNIINGGVECGQGEKAEVADRIGF
YQRYCSIFGISPGQNLDCYNQRPFS

>Cysteine-rich repeat secretory protein (Contig_23860)

MSGNSSFLSLVFYIFLMLHSFAFADPLFQNCSGGNLASNTAYNNLISVNLDLNKTTPYTGFTIVADVTGQKADG
VYGLALCRGDISLQDCATCVGQGSIDSAKLCQNKTSAILWYDHCLYKYTNQDFFGQIDSTNKFDLYSGDVAGNP
TSFNQKTSDFLTLLAKNASSNTPTRFATGVSGPVDGSTTLFGLTQCTWDLSSDACFRCLQEIISGIPTCCSGKL
GARIFYGSCNVRYETYNFIGT

>Lipid transfer protein (Contig_25610 (Fragment))

FDYRFTHHITTTMASSTTLKLICMAFAVVILVAPHAEATFDYDAPNPITAVATTFGVSFCYEYLRTGRGDGLFF
FKPFCCLGLEFIKELAYSSEASLHDTCICLQNLQIFFDISDKYSTSILTFCNITTDYIITRNTDCNKLTL

>β-1, 3-glucanase (BAR13253)

MELSSCASSNDGRYSMAITTLVIGLLLSNVVDTANAIGTCYGMLGNDLPSPQRVVSDYNRYNIKGMRIYGPVSS
LSQALQGSPIAVMVGVPNQDLPGIAMSQSTADSWVQNNIMKYPGVRWKYISAGNEIRPNLDNGGAQYARYVLPA
MQNLQTSVNKFGLRIKVSTALETGICINTYPPSQGQFDPSISGYILPIVKFLAANGSPLFLNVYPYFAYIYTPN
MDIRYALFTSPNVVVQDGQYGYQNLFDAIVDSVYSALEKAGTGMVQIVVTETGWPTAGDKATSIANAQTYNNNL
IQHVKNGTPKRPSKAIETFIFDMYNENLKTPEREKHWGLFKSNGEQKYPIDFN

>S1/P1 nuclease (Contig_14977 (Fragment))

GAQDLASLCSWADQVRFRFHWSSALHFADIQDPNACNFQYDRDCIDTNGVLGRCVVGAIYNYTGQLLDYVNGTE
TGYNYTQSLLFLSHFMGDVHQPLHCGHAVDKGGNTIEVHWYR

>Lipid transfer protein (Contig_29120)
MAGSTMLKLIATSFVCVILVAPQAKAHYTCDPITYVVETVILSPCYGFLKTGKTDALFYNELFCCLAARLVNSF
AYSSPTALKDTCYILENSVSLYKFNPVYGQELFKKCGVNSTYAFDPSLDCSTLKLP
>Hevein-like protein (BAR13255)
MESKGLRFAFLICLVAGAVSQQCGSQAGGQLCPNNLCCSQYGYCGSTDAYCSTANHCQSNCHGGGNTPPSPSSD
IGEGANNVRATYHLYNPQDNGWSLYAVSAYCSTWDGDQSYAWRSKYGWTAFCGPVGPHGQAACGQCLLVTNTAT
GAQATVRIVDQCSNGGLDLDVNVFNAIDTNGQGYAQGHLTVNYQFVNCGD
>GDSL lipase (Contig_30899)
MGTNDWIRNYFIPENPCLRALTYTVDQYADFLVGVFNHSLEVLYKGGCRRFIVYSVPATGCMPTVKTIAGNFSG
KCVDRFNNAAVIHNAKIQQALSGFNQTHDVKTVYVSFYDLLLDATQNPSTYGFTESLIPCCGALLSPRAILCNP
LLLTCPDASKHVYWDSVHPTEAFYELLANKTMEALTLDFF
>Polyvinylalcohol dehydrogenase (FAA01288)
MAPLTKESIFGTCFRIYLVVSCLLATLEGGSAQWPNHGGNLDNTRSPILPQPSINPSKISSLKLKWKFFVGNDI
SATPALWSGNLYFPAWNGVLYAVNALTGVPIWQQNLGQLTGLTPTGTVVNVTVSRATPVVAGDLLIIGIYGPAV
VIAVSRFTGKLVWMTKLDPHALALITMSGTACLDAIYVGVSSLEELLPAEQCCTFRGSMAKLDIKTGKKLWQTY
TVPDNNGKLGGYAGAAIWGSSPSIDVVRNLVYVGTGNLYTAPAAALECQAKQNNETTKPAHPDQCTSPDAHGNS
VLAFDMGTGNIKWYRQLGGYDVFYFACLVPNNPSCPPGPNVDADFGEAPMLLTIQYNGILRDVAVLGQKSGFVW
ALERSSGDIIWFTLAGPGGTEGGGTWGAATDGKRIYTNIVNNNRENFTLAPSTATTTAGGWVALDANTGKILWS
TANPSNDTTNGPVTVVGGVVFAGSVAPNGPFYGMDARTGKVIWTYNTNATIYGGASSSYGCVYIG
>Lipid transfer protein (Contig_30505)
MAGSSVEFIRAFLIGILFFATVASITAEASVCSQVDVVSGECMPYAMGKASSVTSSCCSAVKGLAVLLNTSANK
EQACKCIKAILSNMPGLDINKVKAVPSKCGITDFPLSSC
>Polygalacturonase inhibitor (Contig_23225 (Fragment))
YSPIYGALPSSLTKLKYLTAIRVSGTNLNSTIPSFLGNMTNLQSIDLSSNSLYGTIPSSLGNLVGLNRLDLSNN
HLSGQIPESLGSCKNLDTLDFSNNQLTGDVPKSLSHVNMAYVALGSNQLTGDASHLFQSNTGTLYLYGNRFKFN
FSNVIVDANLAYFDMSNNLVYGGFPPSINDPLFEEFSVSNNRLCGPIPEGGSLQQFQADAFDNNLCLCGRPLPP
CSGS
>Valine-tRNA ligase (Contig_1096 (Fragment))
MSEKTETMELSAEELDKKKKKEEKAREKELKKQKAAQKAEAAKLQVQQPSNASKKSERKNQRRSAEEENADDYI
DPQTSFGEKKLLSRMMAKQYNPSVVEASWYAWWEKSGFFVADAKSSKPPFVIVLPPPNVTGALHIGHALTSAIQ
DTIIRWRQMSGYNTLWVPGMDHAGIATQVVVEKKIMRERKLTRHDLGRDKFVSEVWNWKNEYGGTILRQQRRLG
SSLDWSRECFTMDEKRCLAVTEAFVRLFKEGLIYRDIRLVNWDCVLRTAISDIEVDYQDIKERTLLKVPGYEKD
VEFGVLTSFAYPLEGNLGEIVVATTRVETMLGDTAIAVHPEDTRYQHILGKFAIHPFNGRKLPIIPDPILVDRN
FGTGAVKITPAHDPNDFEVGKRHKLEFINIFTDDGKINGNGGPEFEGMPRFRAREAITKALQEKGLYRGAKNNE
MRLGVCSRSRDVVEPMIKPQWYVNCSSMAKDALNAVMDEENQRIEIIPKQYASDWKRWLENIRDWCVSRQLWWG
HRIPAWYVSLENDERKELGSYNDHWVVARNEEEARKEANERYPGKKFTISQDPDVLDTWFSSGLFPLTVLGWPD
ETEDLKAFYPTSVLETGHDILFFWVARMVMLGMKLGGDVPFRKVYLHPMIRDAHGRKMSKSLGNVIDPLEVING
ITLQGLHKRLEEGNLERAELKTAKAGQEKDFPNGISECGADALRFALVSYTAQSDKINLDIQRVVGYRQWCNKL
WNAIRFAMSKLGDDYTPPTNIVPEILPFCCQWILSVLNKAIGETVASFEAYEFSDAATAVYSWWQFKLCDVYIE
VIKPYFVGSDSSAASSKRFAQDVLWLCLETGLRLLHPFMPFVTEELWQRLPHPKDSVRKESIMISEYPSVVKSW
TNEKVEQDMDLIESAVKALRYLRSLMPVKERHERRPAFALCQTDEVAEIINLHELEICTLANVTSLKVSSSVDD
APVGSTVSVVNE
>Class IV chitinase (Contig_23195 (Fragment))
MISPKYIPNYTPIQTMALSIRTRFAIICVIIIVVFSSSSLAQNCGCDSSLCCSQYGYCGTSDAYCGIGCQEGPC
KYPVNNTKNDVSVPGVVSDAFFNGIIDQAAPTCEGIGFYSRAAFLCAWENYTDF
>S1/P1 nuclease (Contig_3798)
MGSRVGIWVCGAVVFLHLIPSILSWGQHGHYATCKIAEGYISKDAMIAVKQLLPESAGGELAAVCSWPDQIRFH
YPWSGPLHYIDTPDFRCQYDYNRDCHDSAGNKGRCVAGAIYNYTEQLVLGCQNSSFPNSTKYNLTEALMFLAHF
IGDVHQPLHVGFTSDGGGNTIRVRWFRQMSNLHHVWDTSIIDTALKRFYKSDLSDFIREIQLNITGSWADDLVG
WESCASNKTVCPDPYASESISLACRYAYRNATSGSTLGDDYFLSRLPVVEQRLAQGGIRLAATLNRIFSPRLIQ
PHEDTQDCCGSESGITI

>Basic secretory protein (BAR13256)
MARIFLILSSILFLASTQQGQAVIYTVTNNAADTPGGIRFATQIGLDYSRQTLASATAFIWRLFQENSPAERKN
TQHVTLIVESMDGVAYTSADQIHLSANYVQGYSGDVKREITGVLYHEMTHVWQWDGNGQAPGGLIEGMADYVRL
KAGYAPSHWVKPGQGDRWDQGYDVTAYFLDYCDSLRPGFVALLNKMMRSGYSDGFFNQLLGLSVKQLWSNYKAK
YNH
>Serine/threonine-protein kinase (Contig_9565 (Fragment))
MEQYEILEQIGKGAFGSALLVRHKIEKKKYVLKKIRLARQTDRTRRSAHQEMELISKIRNPFVVEYKDSWVERG
CYVCIVIGYCEGGDMAEAIKKANNVHFSEEKLCKWLVQLLMALDYLHTNHILHRDVKCSNIFLTKERDIRLGDF
GLAKLLTSDDLASSVVGTPSYMCPELLADIPYGSKSDIWSLGCCIYEMSALMPAFKAFDMQALINKINKSIISP
LPTMYSGAFRGLVKSMLRKNPELRPSAAELLKHPHLQSHVLKINLKYGNPRRNSLPISDFHNRRRTRFTEPDEV
LEKRRSLGNDRTLNPSVSLTEHYSPSSIKEDEQFSEHLGRRLEDLSVSSVHREVGVDNIAMSKVTNTTRTSRLS
TPAKASATLRRESAPSKFSSPGFNRVSFPVTNAS
>Class III peroxidase (Contig_31040 (Fragment))
LGRFDSLSLTSLNDTSPALTMDLDELTAMFAERNFTQIDMIALFGSHTVGHAHCGTFSNRLYNFSSSSSVDPTL
NPEYAKQLQAECPVNVDPSVEASLDPV
>Calmodulin-binding protein (Contig_21026 (Fragment))
KLPTIRSLTSEIEGVASDRPETGCFCHKLDKLPKFLREIADQIEEFTAQARENFSCPARLGSAVVCERLNHEVT
EAAYPNYLELRILNTLHLPIFTNDEVTGKDGEEISVALVDAMGKVFGSFPEVILNVVVLEGNFNCSNDEKWSKE
DFDMHIVKKRENNALLVGDLQVTLKRGVAKLGDLKFTDNSSFVWSKKFRLGLQVAPSSCKGMRIREAITEPFKV
KDCRVKINGKHDAPQFNDEIWRLKSIGKGGVHHKKLNDKGVATVEDFLRLWVINEQELRQLLLVGMGGMKSKQW
KDLINHAKTCSPGSKLYFVPTANADIGTIILNRFYELSELIIGGRHLSAVSLTDYQKAQARALVKEAYQKKTIM
EYDDSHASQSQHYCNNSITPSMDVVWGVQDIHRPTPVANDGNVPLTECPPCPCLPDHGNSTTMEQPRELQNPCK
HTYTSFHHGECSTFQSHISSFSVDNNLQHSPVGPSSADIEWFWSMEETSSYQDYPPVITGARQHINHNGRFKFK
TVLAHLLSVEIFLKEVRAV
>Class III peroxidase (Contig_46408 (Fragment))
ANRIYNFSATSPVDPTLVPQFAAHLQELCPVNVDPSVRVPLDAVTPDVFDNQYYKNLINHMGLLSVDQMLYSDD
RTRTYVVDWAQNQQHFYDALSAAMVKMGRIGVLSSIDGNIRTH

Figure S1 FASTA file of amino acid sequences of the proteins subjected to the LC-MS/MS analysis. Names in the parentheses indicate the protein ID. The letters starting from "Contig" indicate sequences obtained by *de novo* assembly based on the DRA data DRR051750 (Fukushima *et al.*, 2017) , and the others indicate sequences registered with NCBI.

Cysp1

-400 **GTGTTGAATTTTTATGGATTTTGATAAAAAAAAATTTAATAATGATAGTG**
ARR1AT GT1CONSENSUS

-350 **GGGTTTACGGTAACAATTATGAGAGAAGATAATGAGGTATACTGTATATT**
GT1CONSENSUS

-300 **ATAGCAGGAAATGATGATTTAATATTTTGTAAGATGAAAAATTTACTCAA**
GT1CONSENSUS ARR1AT GT1CONSENSUS

-250 **ATAGAAAAGGATTGTAAGTATATTGAGATATACCAAATAAAAAAAGATTG**
ARR1AT ARR1AT

-200 **CAAAGATTATGGAAAAAGGGAGTATAAGTTTTAGAAGGTTGAGCGATGCC**
ARR1AT
GT1CONSENSUS

-150 **GATTTTCTGGCTCCCTATTCGTCGGCAGTAAATTAATTAGAATAGTGAGG**
ARR1AT GT1CONSENSUS

-100 **ACCAGCTTCGTTTATTACGCAACGAGAAGTTTGTTACTGAACATATTGAT**
GAREAT

-50 **CTTTATGTGCCTATAAATAGAACAGATCACGTTGGTTCCTCATTACCAGT**
CARGCW8GAT GT1CONSENSUS
RHERPATEXPA7 T/GBOXATPIN2
ABRELATERD1

Chi1

-400 **CAGGTATACAGTGACCATATAACAGGAAGATTATGTTTGCCTTTTTAGTT**
ARR1AT

-350 **CTCATTTTTCATGGGCAATCCAATTTTATAACTTCTGGCTTAAGAGCAAC**
GT1CONSENSUS ARR1AT

-300 **CTCTAAATTTTTCCTTGGTCGGCTCGCTGTAGCTTCCAACGTAAAGATAT**
GT1CONSENSUS

-250 **AGCATGACAAATAGGTTGTTAATATTTCGGCCAACCTGTGTGATAAATTT**
GT1CONSENSUS

-200 **CACTCATGCTTTGCCCTCCTAGTGATTTCATTGACCATAGGATCCTAATA**
PREATPRODH ARR1AT WBOXATNPR1

-150 **CAGCATAAAAAATAGTAAGCATTGTCCATGGTCCATGAAATCTATAGATT**
ARR1AT ARR1AT

-100 **ATGAAATTGTCATTAGAAGACTAATTCAATCGAAAATCCCGTGCAGGAAG**
ARR1AT ARR1AT
GT1CONSENSUS

-50 **TCCCTATTTATACACCTCCTGTATCCTAACTTTCACCAGGTCATAATAAA**

Glu1

-400 **TCGGATGGTCTCAGTTGTTATCTTTAATTCAATCAACACATCTTTTGCTG**

SURECOREATSULTR11 MYB2CONSENSUSAT ARR1AT

-350 **TTGAACTCGGATGCAGCTTAGATCAAGGCGAGCCGGCCTGTTCCACTTCT**

-300 **GCAGTGCATGTAAGCACATGACATAGCATTGCAGGACAATCCATGTATCA**

ARR1AT

-250 **GATGGTTTTTCTAGTCGTCAACTGAATGAAAACGTTCGTGGCGTCACAAA**

MYB1AT GT1CONSENSUS MYB2CONSENSUSAT SORLIP1AT

WBOXATNPR1

-200 **GGCGCACATAAATGAAAGTTGGATTCAGTCAAAATGAATACTTTGATGCC**

ARR1AT TBOXATGAPB

WBBOXPCWRKY1 WBOXATNPR1

-150 **ATAACTTGGTATTCTCTTCTTCTAGGCTTGCCTTACAATCTGGGAGTCCA**

ARR1AT

-100 **AGCTCTAGGGACTCAGCTGATTATACCTAGACTATACATTCATCACGTCG**

ARR1AT ANAERO3CONSENSUS

RHERPATEXPA7 ABRELATERD1

-50 **CTATAAATAGCAATGCTTCGTTGCTCGTGTTCTGCAACTACTTTGTCGAC**

CARGCW8GAT TBOXATGAPB

Tlp1

-400 **TTCATCAATCATATGGCGATATTTCATTGCACAAATATAATTACAAAAAA**

ARR1AT ARR1AT

-350 **TCGCGAATTTAAGTTAGAAAAGTTTGAGTTCTTTCAAATAACTTCTCATT**

-300 **GAAAGACCATTATCCTTAGTTTGGTCCCGACCATTCTATACTTCTCTTCA**

GT1CONSENSUS SREATMSD

-250 **CTGAAACATTATTATGATGCCTTTGAACCTTAGGCGTTGCTTAATTCCAG**

CARGCW8GAT

-200 **CTTTAATCTCTGTGATGAGAGTAGTTGCCGTCCTAGACCATGCATAACTA**

ARR1AT ANAERO3CONSENSUS

-150 **AGGTCATTAGACTAAGTCATTGTCCCTCGTTGGTTTAGCAAATTTTTACT**

MYB1AT ANAERO4CONSENSUS

-100 **TGGTCAAGTGTCTGTAGTTAGAGGGAAAACATTGGTCTATAGTCGATTTT**

WBOXATNPR1 GT1CONSENSUS LEAFYATAG ARR1AT

GT1CONSENSUS

-50 **CGTTTTCTATATAAACCAAACTCTCATTCAAAAACATATACAAATATTCC**

MYB1AT MYBPLANT

Hel1

-400 TCAAAATAAAATCTTGAAGGGAATCCTATTTTCCTACCTATGAACTCAAA
ARR1AT ARR1AT GT1CONSENSUS

-350 TTTGAGTTTTTGTGATTTCAATGCCAGGTTTGCCGATATATAGGTACTGG
ARR1AT

-300 AGCGGGTGACTTTTAGCGGCGAAGAGGCGAATATCCGCAAGACGGATCCT
P1BS

-250 TGATCTATCTTCAGAAGCCAAAAATTTTAATGCTGCAAGTATTTTCAACC
GT1CONSENSUS MYBPLANT

-200 AAACAAAACATCATATGTCAGTTAGTATATAGATTAGACTTGGTACCTTC
ANAERO1CONSENSUS MYB2CONSENSUSAT ARR1AT

-150 TTCTAATCTAAGTATACATGTCGACTATTATCATCATAGGTCCATATTTT
ARR1AT GT1CONSENSUS GT1CONSENSUS

-100 CCTTGTGAAGATGTTGTTCGCTCCGTGGCGACTAGTCAAGATTGTAATTC
SORLIP1AT WBOXATNPR1 ARR1AT

-50 TTCCCTATAAATATGGTGACCCTTGTTTCATTCTTTCTATCAACCAGAAA

Figure S2 Upstream sequences and *cis*-DNA elements of *Cysp1*, *Chi1*, *Glu1*, *Tlp1* and *Hel1*. Numbers are relative to transcription start site (+1). PlantPAN3.0 (Chow et al., 2019)-based analyses identified the following elements: ABRELATERD1 (etiolation-responsive element), ANAERO1CONSENSUS (involved in the fermentative pathway), ANAERO3CONSENSUS (involved in the fermentative pathway), ANAERO4CONSENSUS (involved in the fermentative pathway), ARR1AT (ARR1 binding element involved in response to cytokinin, CARGCW8GAT (flower-specific responsive element), GAREAT (GA-responsive element), GT1CONSENSUS (consensus GT-1 binding site in many light-regulated genes), LEAFYATAG (target sequence of LEAFY involved in flowering), MYB1AT (MYB recognition site involved in dehydration-response), MYB2CONSENSUSAT (MYB recognition site involved in dehydration-response), MYBPLANT (plant MYB binding site involved in anthocyanin biosynthesis), P1BS (PHR1-binding sequence found in the upstream regions of phosphate starvation responsive genes from several plant species), PREATPRODH (pro- or hypoosmolarity-responsive element), RHERPATEXPA7 (root hair-specific element), SORLIP1AT (sequence over-represented in light-induced promoter), SREATMSD (sugar-repressive element), SURECOREATSULTR11 (core of sulfur-responsive element), TBOXATGAPB (light-responsive element), T/GBOXATPIN2 (involved in jasmonate signaling), WBBOXPCWRKY1 (pathogen/elicitor response element), WBOXATNPR1 (involved in SA-response).

Table S1 Summary of iBAQ values in the Asp-N digestion

Protein name [a]	iBAQ value	Abundance rank
Thaumatin-like protein (BCF79772)	2.09E+05	1
Lipid transfer protein (BAW35429)	1.22E+05	2
S-like RNase (BAE16663)	1.10E+05	3
Unknown (Contig_14 (Fragment))	3.60E+04	4
Cysteine protease (BAW35427)	2.54E+04	5
Lipid transfer protein (Contig_35636)	2.15E+04	6
Unknown (Contig_1360)	1.94E+04	7
Class I chitinase (BAR13254)	1.58E+04	8
Cysteine-rich repeat secretory protein (Contig_23860)	9.17E+03	9
Lipid transfer protein (Contig_25610 (Fragment))	7.38E+03	10
Hevein-like protein (BAR13255)	5.83E+03	11
β-1, 3-glucanase (BAR13253)	5.05E+03	12
Polyvinylalcohol dehydrogenase (FAA01288)	4.69E+03	13
Lipid transfer protein (Contig_29120)	3.95E+03	14
GDSL lipase (Contig_30899)	3.65E+03	15
Lipid transfer protein (Contig_30505)	2.57E+03	16
Polygalacturonase inhibitor (Contig_23225 (Fragment))	1.92E+03	17
Class IV chitinase (Contig_23195 (Fragment))	1.38E+03	18
Basic secretory protein (BAR13256)	9.31E+02	19
Class III peroxidase (Contig_31040 (Fragment))	8.82E+02	20
Class III peroxidase (Contig_46408 (Fragment))	6.56E+02	21

[a] Names in the parentheses indicate the protein ID. The letters starting from "Contig" indicate sequences obtained by *de novo* assembly based on the DRA data DRR051750 (Fukushima *et al.*, 2017) , and the others indicate sequences registered with NCBI.

Table S2 Summary of iBAQ values in the chymotrypsin digestion

Protein name [a]	iBAQ value	Abundance rank
Thaumatin-like protein (BCF79772)	6.43E+04	1
Unknown (Contig_14 (Fragment))	5.53E+04	2
S-like RNase (BAE16663)	3.09E+04	3
Cysteine protease (BAW35427)	1.89E+04	4
Lipid transfer protein (BAW35429)	1.53E+04	5
Unknown (Contig_1360)	1.08E+04	6
Class I chitinase (BAR13254)	1.05E+04	7
Cysteine-rich repeat secretory protein (Contig_23860)	9.45E+03	8
Lipid-transfer protein (Contig_29120)	4.90E+03	9
S1/P1 nuclease (Contig_14977 (Fragment))	2.61E+03	10
GDSL lipase (Contig_30899)	1.54E+03	11
Hevein-like protein (BAR13255)	1.32E+03	12
Polyvinylalcohol dehydrogenase (FAA01288)	8.34E+02	13
Valine-tRNA ligase (Contig_1096 (Fragment))	3.00E+02	14
S1/P1 nuclease (Contig_3798)	1.17E+02	15
Serine/threonine protein kinase (Contig_9565 (Fragment))	1.15E+02	16
Calmodulin-binding protein (Contig_21026 (Fragment))	5.75E+01	17

[a] Names in the parentheses indicate the protein ID. The letters starting from "Contig" indicate sequences obtained by *de novo* assembly based on the DRA data DRR051750 (Fukushima *et al.*, 2017) , and the others indicate sequences registered with NCBI.

Table S3 PCR primers used in the Real-Time PCR

Gene name	Primer sequence (5' to 3')
actin	F: TGAGGCACCACTCAACCCTA
	R: AGAGAAAGCACCGCCTGAA
eIF4A	F: ATTGGCCCGTGGTATTGATGTC
	R: TTGATTGCCACACCCTTTCTCC
TIP41	F: TTGGCTGCAAAGGAACCCATTC
	R: AACCAACCACTCGGCATAACTC
Cysp1	F: CAGAGGAGTCACCACCGAAG
	R: CCCGTTCGCTATTGTTTGGA
da-I	F: ATGGAACAAGCACGGAACCT
	R: TAGGCTGGCAAGGGTATTGG
Chi1	F: TTGTGCCGCTTCTTTCAGG
	R: CAGCATAGCCCGTTAGGACA
Glu1	F: GGACATCAGGTATGCTCTATTCACC
	R: TTCCAGTTCCCGCCTTCTC
Tlp1	F: GGGTCGTACAGGATGCACAGT
	R: CCGCCAGAGTATTAGGAGGTTG
Hel1	F: GCGACGGTAAGGATTGTGGA
	R: CATAGCCTTGTCCGTTGGTG
DaMET1	F: GGTGCTGATTGGCGTGA
	R: TGGCGGTGTTTGGGAGA
DaCMT3	F: TCTCCCTCAATTTCGCCTTC
	R: GGGACGACACCCCTCATAAC
DaDRM2	F: AGTAGGACAGACCGCTACAAATCAC
	R: CCAATGCCTGAGAAGAGAGACA
DaROS1	F: AAAGAAAGACTGCCCCATCAC
	R: TGTTGTTCATTCCCCTCTCCT
DaDME	F: TCCACCACAGAAATCGAAGG
	R: GGTGAAGCGTTAGCAGTCGT

Table S4 PCR primers used in the bisulfite sequencing

Gene name		Primer sequence (5' to 3') [a]
Cysp1	F:	ACACTCTTTCCCTACACGACGCTCTTCCGATCTAATTATGAGAGAAGATAATGAGGTA
	R:	GTGACTGGAGTTCAGACGTGTGCTCTTCCGATCTTCTTTTCACCATTRATRRTTCAT
Chi1	F:	ACACTCTTTCCCTACACGACGCTCTTCCGATCTGAAGTATTATYAYAGTATTGTTGTG
	R:	GTGACTGGAGTTCAGACGTGTGCTCTTCCGATCTARAAATCTTCATRTTTTTTAATCCC
Glu1	F:	ACACTCTTTCCCTACACGACGCTCTTCCGATCTTTTTGYTGTTGAAYTYGGATG
	R:	GTGACTGGAGTTCAGACGTGTGCTCTTCCGATCTCCATTRAATATCTTCCATCATT
Tlp1	F:	ACACTCTTTCCCTACACGACGCTCTTCCGATCTTYATTTYTTGGTTTAATATTGTGAT
	R:	GTGACTGGAGTTCAGACGTGTGCTCTTCCGATCTCAATTRCTTRTTATATACCTTCTAT
Hel1	F:	ACACTCTTTCCCTACACGACGCTCTTCCGATCTTTAGGATGGTYYATTAYTGATTAAG
	R:	GTGACTGGAGTTCAGACGTGTGCTCTTCCGATCTCARATCAARAATRCAAACCTC

[a] The underlined portions indicate overhangs.

Table S5 C5-MTases and DNA demethylases identified in *D. adelae*

Name	Gene		Length (aa)	kDa	pI
	Name	Accession number			
DNA (cytosine-5)-methyltransferase 1	*DaMET1*	YAAA01000001	1579	177.2	5.70
DNA (cytosine-5)-methyltransferase CMT3	*DaCMT3* [a]	YAAA01000002	765	-	-
DNA (cytosine-5)-methyltransferase DRM2	*DaDRM2*	YAAA01000003	613	68.5	4.97
DNA glycosylase/AP lyase ROS1	*DaROS1*	YAAA01000004	1902	211.0	5.84
Transcriptional activator DEMETER	*DaDME*	YAAA01000005	2005	223.2	6.95

[a] Sequence is partial.
